# Frailty syndrome and cognitive impairment in older adults: systematic review of the literature*

**DOI:** 10.1590/1518-8345.3189.3202

**Published:** 2019-10-28

**Authors:** Karen Miyamura, Jack Roberto Silva Fhon, Alexandre de Assis Bueno, Wilmer Luis Fuentes-Neira, Renata Cristina de Campos Pereira Silveira, Rosalina Aparecida Partezani Rodrigues

**Affiliations:** 1 Universidade de São Paulo, Escola de Enfermagem de Ribeirão Preto, PAHO/WHO Collaborating Center for Nursing Research Development, Ribeirão Preto, SP, Brazil.; 2 Scholarship holder at the Coordenação de Aperfeiçoamento de Pessoal de Nível Superior (CAPES), Brazil.; 3 Scholarship holder at the Conselho Nacional de Desenvolvimento Científico e Tecnológico (CNPq), Brazil.; 4 Universidad de San Marcos, Escuela de Nutrición, Lima, Peru.

**Keywords:** Aged, Frailty, Cognition, Cognitive Aging, Meta-analysis, Review, Idoso, Fragilidade, Cognição, Envelhecimento Cognitivo, Metanálise, Revisão, Anciano, Fragilidad, Cognición, Envejecimiento Cognitivo, Metaanálisis, Revisión

## Abstract

**Objective:**

to synthesize the knowledge about the association of frailty syndrome and cognitive impairment in older adults.

**Method:**

the Joanna Briggs Institute’s systematic review of etiology and risk factors was adopted. The search for the studies was conducted by two independent reviewers in the databases MEDLINE, Embase, CINAHL and LILACS and by manual search was performed by tow reviewers independently. The measures of association Odds Ratio and Relative Risk were used in the meta-analysis. The *software* R version 3.4.3 and the meta-analysis package Metafor 2.0 were used for figure analysis.

**Results:**

three studies identified the association of frailty syndrome and cognitive impairment through Odds Ratio values show that frail older adults are 1.4 times more likely to present cognitive impairment than non-frail older adults. Four studies analyzed the association through the measure of Relative Risk and found no statistical significance, and four studies used mean values.

**Conclusion:**

despite of the methodological differences of the studies and the lack of definition of an exact proportion in the cause and effect relationship, most studies indicate Frailty Syndrome as a trigger for Cognitive decline.

## Abstract (pt)

**Objetivo:**

sintetizar o conhecimento sobre a associação entre a síndrome da fragilidade e o comprometimento cognitivo do idoso.

**Método:**

adotou-se o referencial do *The Joanna Briggs Institute* de revisão sistemática de etiologia e fatores de risco. A busca dos artigos nas bases de dados MEDLINE, Embase, CINAHL e LILACS e a busca manual foi feita por dois revisores de forma independente. Para a metanálise, empregaram-se as medidas de associação *Odds Ratio* e do *Relative Risk*; para a análise dos gráficos, foram empregados o *software* R (versão 3.4.3) e o pacote de metanálise *Metafor* 2.0.

**Resultados:**

na associação entre a síndrome da fragilidade e o comprometimento cognitivo, três estudos identificaram associação por meio da medida do *Odds Ratio*. Mostrou-se que os idosos frágeis têm 1,4 mais chance de apresentar comprometimento cognitivo em relação aos não frágeis, em quatro estudos foi possível estabelecer a relação mediante a análise do *Relative Risk* e não houve significância estatística, em quatro estudos utilizaram a média.

**Conclusão:**

apesar das diferenças metodológicas usadas nos estudos e a não definição da uma proporção exata na relação causa-efeito, a maioria dos estudos indica que a Síndrome de Fragilidade desencadeia o Comprometimento Cognitivo.

## Abstract (es)

**Objetivo:**

sintetizar el conocimiento sobre la asociación entre el síndrome de fragilidad y el deterioro cognitivo del adulto mayor.

**Método:**

se adoptó el referencial del *The Joanna Briggs Institute* de revisión sistemática de etiología y factores de riesgo. Se realizó una búsqueda en las bases de datos MEDLINE, Embase, CINAHL, LILACS y la búsqueda manual fue realizada por dos revisores de forma independiente. Para el meta-análisis se empleó la medida de asociación *Odds Ratio* y *Relative Risk*. Para el análisis de los gráficos se utilizó el *software* R versión 3.4.3 y el paquete de meta-análisis *Metafor* 2.0.

**Resultados:**

en la asociación entre el síndrome de fragilidad y deterioro cognitivo, tres estudios identificaron asociación por intermedio de la medida de *Odds Ratio*. Se demostró que los adultos mayores frágiles tienen 1,4 más posibilidades de presentar deterioro cognitivo comparado con los no frágiles, en cuatro estudios fue posible establecer la relación mediante el análisis del *Relative Risk* y no hubo relevancia estadística, y cuatro utilizaron la media.

**Conclusión:**

a pesar de las diferencias metodológicas utilizadas en los estudios y la falta de definición de una proporción exacta en la relación de causa y efecto, la mayoría de los estudios indican que el síndrome de fragilidad desencadena un deterioro cognitivo.

## Introduction

The World Population Ageing report, published by the United Nations Population Division estimates that between 2015 and 2030, the number of people in the world aged 60 years or over is projected to grow by 56 per cent, from 901 million to 1.4 billion , and by 2050, the global population of older adults is projected to reach nearly 2.1 billion^(^ 1 ^-^ 2 ^)^.

It is fundamental to recognize the demographic growth as a current and relevant process for society in order to understand the specific needs of older adults, since the aging process involves changes in the functions of the human organism.

These changes can be structural and functional and can modify the person’s abilities in their activities of daily living and lead to loss of independence and autonomy^(^ 3 ^)^. Among the various concerns within this field, two themes have been the subject of several debates and studies: Frailty syndrome and Cognitive impairment.

During the aging process, there is a gradual and cumulative decline in physiological reserve, influenced by underlying genetic and environmental factors^(^ 4 ^)^. This disorder of many physiological systems is known as frailty, a syndrome that affects a large number of older adults. Frailty is a clinical condition in which there is an increase in an individual’s vulnerability, leading to several consequences, such as increased dependency, and even mortality when the person is exposed to stressors^(^ 5 ^-^ 6 ^)^.

More broadly, frailty can be defined as a medical syndrome with multiple causes, characterized by diminished strength, muscular endurance and reduced quality of physiological function, factors that increase an individual’s vulnerability for developing increased dependency or death^(^ 6 ^)^.

Another condition associated with the aging process is cognitive impairment. It is a lifelong process of change in cognitive functioning. Despite not being considered a disease, or a quantifiable level of function and it is a gradual and ongoing process^(^ 7 ^)^. Recent findings indicate that this condition, despite being a natural consequence of this process, can be reversed or modified^(^ 8 ^)^.

Current debates about the incidence of frailty constantly include the topic of cognitive impairment. Its incidence is directly proportional to the increase in age and there is evidence of a biological substrate of frailty that may promote or accelerate cognitive decline^(^ 9 ^)^. This hypothesis reinforces the argument that frailty syndrome and cognitive decline share the same pathophysiological mechanisms.

Thus, frailty syndrome and cognitive impairment have been increasingly studied, because they are considered a public health issue, in the sense that their early detection has a direct impact on health outcomes^(^ 10 ^)^.

Given the importance of the topic, associated with an increased population aging and greater life expectancy, a systematic review was conducted to identify the relationship between frailty syndrome and cognitive impairment. The researchers started the discussion on the topic of physical frailty, however, in the nursing practice, cognitive impairment was also identified as a fragility of older adults in the development of their daily activities, impairing spatial function, temporality, calculations, sentence construction, self-care and affection. These aspects are essential for the development and insertion of older adults in their social environment. Thus, the present study aimed to search the literature for important evidence, with the purpose of contributing to the practice of nurses/health professionals regarding the evaluation and follow-up of older adults in different health care settings. Therefore, the objective of this study was to synthesize the knowledge about the association between frailty syndrome and cognitive impairment in older adults through a systematic review.

A preliminary study protocol was elaborated with the objective of finding a systematic approach to be employed in the review. This enabled a transparent process, methodological rigor and reduced the possibility of bias in the final report. The protocol elaborated guided a prior search in the main databases to find out if there were any proposed or conducted systematic review that answered the present guiding question^(^ 11 ^)^. Other reviews found discussed the proposed theme, but not with the emphasis that guides this study: to identify the association between cognitive impairment and frailty syndrome in older adults. In this protocol, the objectives, criteria and methods were previously specified.

## Method

This is a systematic review of etiology and risk factors with meta-analysis, based on the recommendations of The Joanna Briggs Institute (JBI). The model adopted aimed to analyze the association between certain factors and the development of a disease, condition or other health outcome, following a structured process, with a rigorous method, to ensure that the results achieved are reliable and meaningful. This review followed eight steps, namely: 1) title of the review; 2) objective and guiding question; 3) introduction (background); 4) inclusion criteria; 5) methods (search strategy, critical appraisal, selection of studies and synthesis of data); 6) results; 7) discussion; and 8) conclusion and recommendations^(^ 11 ^)^.

The first step of the review was choosing the title, which presented the main elements of the guiding question, aligned with the objectives and the inclusion criteria. Then, a protocol was structured so the whole process of this systematic review would be guided by the objectives and methods.

The objective and the guiding question were elaborated according to the PEO model, with P = Population (older adults); E = Exposure of interest (frailty syndrome) and O = Outcome (cognitive impairment^(^ 12 ^)^.Based on this strategy, it was possible to construct the critical thinking about the topic and formulate the following question: *What knowledge is available in literature on the association between frailty syndrome and cognitive impairment in older adults?*

Inclusion and exclusion criteria:

- Inclusion criteria: Studies with older adults ≥ 60 years about frailty syndrome and cognitive impairment, regardless of gender, ethnicity, social condition, presence of comorbidities, place of residence and in different settings (hospital, home and nursing home for older adults); Observational studies with prospective follow-up, in which older adults were evaluated at different times, published in Portuguese, English or Spanish assessing frailty syndrome and cognitive impairment, and with limitation regarding date of publication.
- Exclusion criteria: Literature review studies; thesis and dissertations; book chapters; technical reports and letters from the publisher.

The search for studies was performed in the databases: National Center for Biotechnology Information (NCBI/PubMed), Cumulative Index to Nursing and Allied Health Literature (CINAHL), Latin-American and Caribbean Center on Health Sciences Information (LILACS) and Excerpta Medica Database (EMBASE).

The search strategy combined the controlled vocabularies and the Keywords, according to the indications from each database. The Medical Subject Headings (MeSH) controlled vocabulary was used to search for articles in PubMed; the Heading-MH was consulted for the CINAHL database; the Embase Subject Headings (EMTREE) was used in the EMBASE; and the Health Sciences Descriptors (DeCS) were used in the LILACS database.

The keywords were established based after readings related to the research subject. In order to extend and direct the search, the controlled vocabularies and keywords were combined with Boolean operators (Figure 1).

**Figure 1:** Controlled vocabularies and Keywords used according to the PEO model^‖^ and use of Boolean operators, in July 2018

	Population (P)	Exposuse of interest (E)	Outcome (O)
Database	Older adults	Frailty syndrome	Cognitive decline
PubMed* (MeSH)	“aged”[Mesh] OR “aged, 80 and over”[Mesh] OR “older people” OR “elderly people”	Frail*	“cognitive frailty” OR “cognitive impairment” OR “cognitive decline” OR “cognition disorders”[Mesh]
EMBASE^†^ (EMTREE)	‘aged’/exp OR ‘very elderly’/exp OR ‘frail elderly’/exp	Frail*	‘cognitive defect’/exp OR ‘mild cognitive impairment’/exp OR ‘cognitive decline’/exp OR ‘cognitive impairment no dementia’/exp)
CINAHL^‡^ (Headings-MH)	MH “aged” OR MH “aged, 80 and over” OR “older person”	Frail*	MH “cognition disorders” OR “cognitive impairment” OR “cognitive decline” OR “cognitive frailty”
LILACS^§^ (DeCS)	“idoso” OR “aged” OR “anciano” OR “idoso de 80 anos ou mais” OR “aged, 80 and over” OR “anciano de 80 o más años” OR “idoso fragilizado” OR “frail elderly” OR “anciano frágil”	“frágil” OR “frail” OR “frágil”	“disfunção cognitiva” OR “cognitive dysfunction” OR “disfunción cognitiva”

* PubMed = National Center for Biotechnology Information;

National Center for Biotechnology Information;

† EMBASE = Excerpta Medica Database;

‡ CINAHL = Nursing and Allied Health Literature;

§ LILACS = Latin-American and Caribbean Center on Health Sciences Information;

‖ PEO = (P-Population; E-Exposure of interest; O-Outcome)

The Boolean operator OR, AND was used for addition and for restriction, respectively. The asterisk was used after the word “frail”, because it represents the exposure of interest and with the purpose of expanding the scope of search. In addition, the search was performed using identified and extended vocabulary and without using filters from the databases, in order to obtain a significant sample with a lower risk of loss. This strategy explains the small number of studies selected in comparison with the sample obtained. This way, the final combination of the database search strategy was elaborated.

At this stage of the review, the Rayyan application, developed by the Qatar Computing Research Institute (QCRI)^(^ 13 ^)^, was used as an auxiliary tool for archiving, organizing and selecting articles. The final search in the four selected databases was carried out on July 15, 2018, and 3,284 studies were identified. In addition, two studies from a manual search were included, totaling 3,286 studies. After the identification of the articles in the databases, the titles and abstracts were read according to the Preferred Reporting Items for Systematic Reviews and Meta-Analyses (PRISMA^(^ 14 ^)^. The sample was selected by two independent and blinded reviewers. After this selection, a third reviewer, along with the other two was responsible for analyzing the articles and deciding on the inclusion or exclusion of each one, especially those that caused disagreement. The manual search was performed after the selection of the third reviewer and was based on the references of the selected articles.

The quality assessment of studies was the necessary process of establishing internal validity, by verifying possible biases and the reliability of the evidence^(^ 15 ^)^. In this study, the methodological quality assessment was performed by two independent reviewers, using the Methodological Index for Non-Randomized Studies (MINORS)^(^ 16 ^)^. This instrument contains eight items for non-comparative studies: 1) A clearly stated aim; 2) Inclusion of consecutive patients; 3) Prospective collection of data; 4) Endpoints appropriate to the aim of the study; 5) Unbiased assessment of the study endpoint; 6) Follow-up period appropriate to the aim of the study; 7) Loss to follow-up less than 5%; and 8) Prospective calculation of the study size. Each item was rated from 0 to 2, which means: the score 0 indicates that the information was not reported, 1 indicated that the information was inadequately reported, and 2 that the information was adequately reported^(^ 16 ^)^.

The data extraction occurred in two phases: in the first one, the data from the articles included in the study were extracted by the reviewer (researcher); in the second, another data extraction was carried out by a second reviewer. The data extracted referred to specific information related to the research question and the objective of the review, such as: author(s); year of publication; journal; language; country; title; objective(s); population; context; type of study/method; sample size; focus on cognitive function and frailty syndrome; and results of the analysis performed. The data extracted were grouped in tables according to the values of the measures of association between the studied variables. The data extracted aimed to characterize the study in its general aspects and the method used for the research, along with the respective results.

A narrative synthesis was used to present the results. This approach is characterized by the descriptive analysis of the quantitative data and meta-analysis of the measures of Odds Ratio (OR) and Relative Risk (RR). The analysis of the graphs was performed using the software R version 3.4.3 and the meta-analysis package Metafor 2.0. The forest plots show the association measures (OR and RR) on the X-axis and the Confidence Interval (CI) within the estimated limit of ± 1.96 SE, where SE is the Standard Error.

To reduce the effect of confounding factors, risk estimates were adjusted for the multivariate model of each study and the fixed effects model was adjusted according to heteroskedasticity and weighted least squares. The Cochran’s Q test was used to assess heterogeneity of the results of each risk measure (OR and RR). There were no significant changes (p <0.10) in the heterogeneity between studies, and a fixed effects model was applied. According to the general linear fixed effects model, all studies estimated the same effect size, so inferences can be drawn from all studies, based on the amount of information extracted in this analysis.

The discussion, conclusion and recommendations are based in the analysis found in the final part of this study.

Considering that this study used studies found in public and free databases of the scientific literature, there was no need for processing in the Research Ethics Committee, according to National Health Council Resolution 466/2012, and the actual ethical standards^(^ 17 ^)^.

## Results

**Figure 2:**
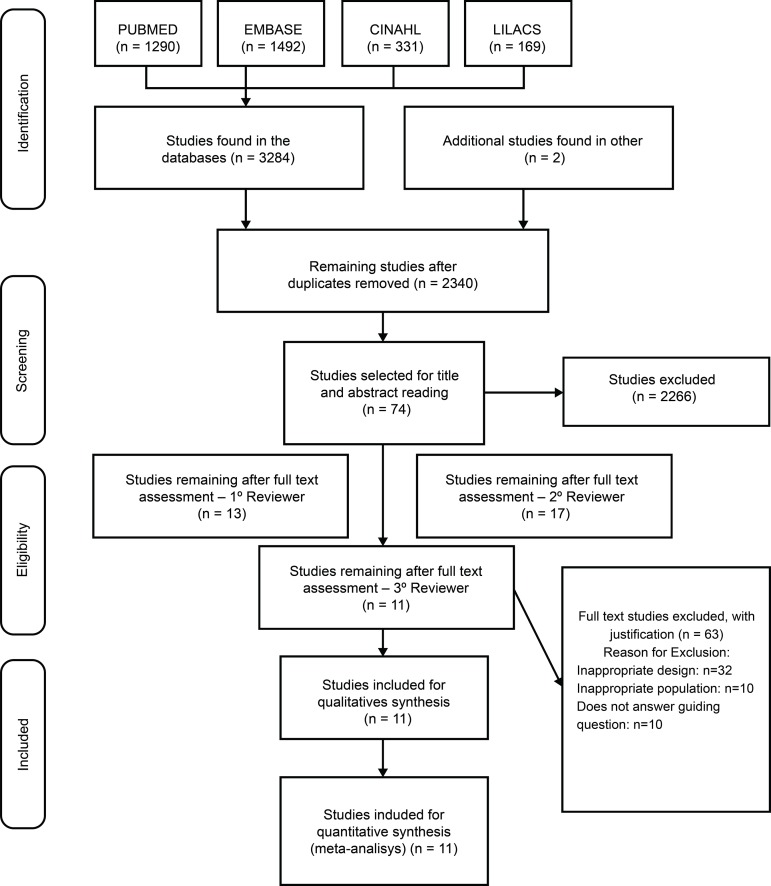
PRISMA flowchart for the selection of studies. Ribeirão Preto, SP, Brazil, 2018

Of the 3,286 studies identified in the four databases and in the manual search, 946 duplicates were excluded and 2,340 were selected to read titles and abstracts. Among these, 2,266 studies were excluded because they did not meet the inclusion criteria and, after evaluation of the studies, 74 studies were selected for reading in full. After this step, 63 studies were excluded, resulting in 11 articles included in this study (Figure 2).

The publication dates of the eleven articles included ranged from 2008 to 2018. All studies were published in English and had a prospective observational longitudinal design. The follow-up duration of the studies ranged from one to ten years, and two to six evaluations were conducted according to follow-up duration, that is, the longer the time, the larger the number of evaluations performed.

In the eleven studies included, the researchers analyzed the association between frailty syndrome and cognitive impairment in older adults, with a total sample of 12,656 non-frail participants with no cognitive decline in baseline assessment. All the older adults lived in houses. Among the participants, 3,445 (27.22%) were in the Asian continent; 3,834 (30.29%) were in the United States; 2,305 (18.27%) were in Canada; 2,890 (22.84%) were in Europe and 182 (1.44%) were in Brazil. Half of the studies described age by mean and standard deviation (SD)^(^ 18 ^-^ 22 ^)^, but were presented according to frailty (non-frail, pre-frail or frail), cognitive impairment as assessed by the Mini-Mental State Examination (MMSE <or> 21) or gender (female or male).

Of the total of 12,656 older participants, one of the studies^(^ 10 ^)^ did not present data related to gender and another one assessed only women^(^ 23 ^)^. Thus, the analysis of this variable was performed with 10,612 older adults, most (59.3%) were female, with a range from 44.7%^(^ 24 ^)^ to 87.4%^(^ 25 ^)^.

In the 11 studies assessed, the authors proposed different types of analysis, according to Table 2. The Logistic Regression Model^(^ 19 ^)^, the General Estimation Equation Model^(^ 26 ^)^, and two types of analysis, General Estimation Equations and General Linear Mixed Models^(^ 27 ^)^were used to identify OR values. In the identification of the topics studied^(^ 19 ^-^ 27 ^)^, cognitive impairment was proposed, while one study^(^ 26 ^)^ established frailty syndrome as dependent variable.

Regarding OR values, one study^(^ 27 ^)^ showed association between the two variables, (OR: 1.27; 95%CI 1.07-1.52), indicating that there are 1.27 more chances of frail individuals having cognitive decline (MMSE <21) in comparison to non-frail individuals and over a period of ten years. According to the CI, the frailty syndrome is considered a risk factor for cognitive decline. Another study^(^ 19 ^)^found (OR: 2.28; 95%CI 1.02-5.08), thus showing frailty syndrome as a risk factor for cognitive impairment, in such a way that frail individuals were 2.28 times more likely to have cognitive decline in comparison to non-frail older adults (using the Montreal Cognitive Assessment (MoCA) <26).

Two studies analyzed RR, the Fisher’s test^(^ 25 ^)^ and Multinomial Logistic Regression^(^ 20 ^)^. The RR were associated (RR: 4.6; 95%CI 1.93-11.2)^(^ 25 ^)^. The CI above one indicates that the exposure (frailty syndrome) can be interpreted as a “risk factor” for the endpoint studied (cognitive decline). On the other hand, other authors^(^ 20 ^)^ used the different domains of cognitive decline evaluation, associating these variables with frailty. Processing speed was the only domain with statistically significant association, with the value (RR: 0.26; 95%CI 0.16-0.42). In this case, as the CI is below one, exposure (processing speed) is interpreted as a “protective factor” for the endpoint frailty syndrome.

It was also verified that two studies^(^ 22 ^-^ 23 ^)^ used the Hazard Ratio (HR) as a measure of association between the study variables. In the evaluation of cognitive impairment of the studies analyzed, the Kaplan-Meier Survival Analysis^(^ 23 ^)^ showed that in the domain of executive functioning it was related to frailty, with 3.3 (95% CI: 1.4 to 7,6). On the other hand, other authors^(^ 22 ^)^ who used Logistic Regression found no association between variables.

Four studies used mean as a measure of association between the variables frailty and cognitive impairment^(^ 24 ^)^, and adopted Multiple Linear Regression^(^ 10 ^,^ 21 ^)^ and Poisson Regression^(^ 18 ^)^.

Authors of one study^(^ 24 ^)^ categorized participants according to gender, cognitive decline and the domains of the frailty scale. In men and women, handgrip strength was associated with cognitive declines, with values (HR:0.197; 95%CI 0.037-0.354) and (HR:0.233; 95%CI 0.086-0.375), respectively.

Other researchers evaluated frailty syndrome and used three scale: in the Comprehensive Geriatric Assessment (FI-CGA; 1.01; 95%CI 0.93-1.07); in the Frailty Phenotype (1.02; 95%CI 0.97-1.09); and in the Clinical Frailty Score (CFS) (1.01; 95%CI 0.95-1.08)^(^ 21 ^)^.

Other analysis models used^(^ 17 ^,^ 28 ^)^ were Bivariate Random 0odels and Poisson’s. The authors found that the correlation coefficient between the frailty phenotype and to evaluate the cognition of the older adults was (-0.73; p <0.001, 19 tests) indicating a strong correlation between the studied variables.

A study conducted in Spain used three frailty scales and observed a mean reduction in the Frailty Phenotype (FP; 2.19 points), Frail Trail Scale (FTS; 1.97 points) and Frailty Index (FI; 1.97 points) for each point of the MMSE^(^ 10 ^)^.

In the eleven studies analyzed, the independent variable considered was cognitive impairment^(^ 18 ^,^ 20 ^,^ 23 ^,^ 26 ^)^ or frailty^(^ 10 ^,^ 19 ^,^ 22 ^,^ 24 ^-^ 25 ^,^ 27 ^)^ (Figure 3).

**Figure 3:** Characteristics of the studies included in the systematic review, according to author, type of analysis, results (Odds Ratio, Relative Risk, Hazard Ratio and Mean) and variables. Ribeirão Preto, SP, Brazil, 2018

Author and Year	Type of analysis	Results	Dependent variable	Independent variable
Odds Ratio (OR)				
Chen et al., 2017(19)	Logistic regression model	2.28 (1.02-5.08)	Cognitive decline	Frailty syndrome
Raji et al., 2010(26)	General estimation equation model	1.04 (0.75-1.44)	Frailty syndrome	Cognitive decline
Samper-Ternent et al., 2008(27)	General linear mixed models General estimation equations	1.27 (1.07-1.52)	Cognitive decline	Frailty syndrome
Relative Risk (RR)				
Gale et al., 2017(20)	Multinomial logistic regression	1) 0.95 (0.56-1.63) 2) 0.75 (0.48-1.15) 3) 0.26 (0.16-0.42) 4) 0.92 (0.69-1.24)	Frailty syndrome	1) Visuospatial ability 2) Memory 3) Processing speed 4) Crystallized cognitive ability
Alencar et al., 2013(25)	Chi-square test Fisher’s test	4.6 (1.93-11.2)	Cognitive decline	Frailty syndrome
Hazard Ratio (HR)				
Gross et al., 2016(23)	Kaplan-Meier survival analysis	1) Psychomotor speed 2.0 (0.8-5.3) 2) Executive functioning 3.3 (1.4-7.6) 3) Memory 1.0 (0.3-3.4) 4) Delayed memory 1.8 (0.5-5.6)	Time to Onset of Frailty	1) Psychomotor speed 2) Executive functioning 3) Memory 4) Delayed memory
Montero-Odasso et al., 2016(22)	Logistic regression	0.2 (0.0-1.5)	Cognitive decline	1) Weight loss 2) Poor grip strength 3) Exhaustion 4) Slow walking speed 5) Low physical activity
Mean				
Rosado-Artalejo et al., 2017(10)	Multiple linear regression	1) Frailty phenotype: -2.19 2) Frailty Trait Scale: -0.92 3) Frailty Index: -1.39	Cognitive decline	Frailty syndrome evaluated by 3 scales (Frailty phenotype, Frailty Trait Scale e FI*)
Buchman et al., 2014(18)	Bivariate random coefficient models Pearson coefficient	Fried phenotype 19 tests 0.07 (SD=0.63)^†^	Frailty	Cognitive function
Mitnitski et al., 2011(21)	Poisson Regression (CI 95%)^‡^	1) FI - CGA: 1.01 (0.93-1.07)^§^ 2) Frailty phenotype: 1.02 (0.97-1.09) 3) CFS: 1.01 (0.95-1.08)^‖^	Cognitive decline	Frailty syndrome evaluated by 3 scales (FI-CGA^§^; Frailty phenotype e CFS^‖^)
Auyeung et al., 2011(24)	Multiple linear regression (CI 95%)^‡^	Female 1) 0.197 (0.037-0.354) 2) -0.059 (-0.214-0.095) 3) 0.020 (-0.142-0.182) 4) 0.055 (-0.105-0.215) 5) -0.042 (-0.209-0124) Male 1) 0.233 (0.086-0.375) 2) -0.233 (-0.373 - -0.088) 3) 0.162 (0.013-0.309) 4) 0.140 (-0.007-0.287) 5) 0.033 (-0.114-0.181)	Cognitive decline	1) Handgrip strenght 2) Chair-stand test 3) Step length 4) Timed walk

* Frailty Index;

† Standard Deviation;

‡ 95% Confidence Interval;

§ Frailty Index = Comprehensive Geriatric Assessment;

Clinical Frailty Scale

**Figure 4:**
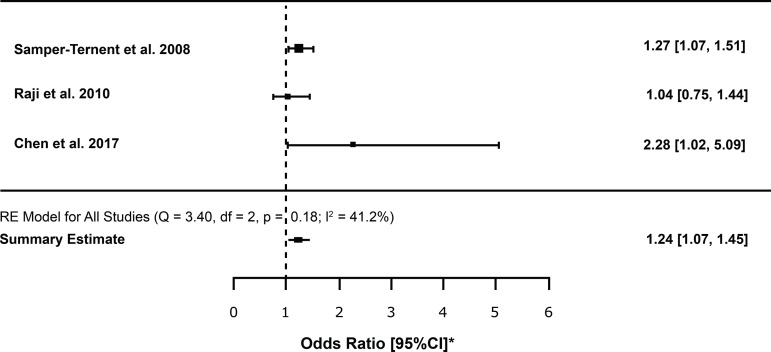
Meta-analysis of Odds Ratio comparing frailty syndrome and cognitive impairment. Ribeirão Preto, SP, Brazil, 2018 *95% Confidence Interval

Regarding the values of the studies that used OR as measure of association, we observed that two studies^(^ 19 ^,^ 27 ^)^results did not cross the vertical line, which means there is association. The data from study did not present statistical significance^(^ 26 ^)^ (Figure 4).

In the meta-analysis, the diamond is on the right side of the vertical line, meaning that the frail older person is 1.24 times more likely to have cognitive decline in comparison to the non-frail individual (statistically significant, with p<0.005).

A moderate heterogeneity between studies (I^2^ =41.2%). However, I^2^ were not statistically significant (p=0.18).

**Figure 5:**
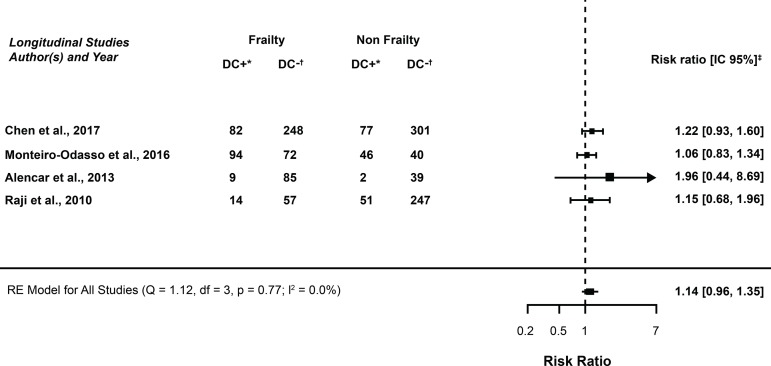
Meta-analysis of the association between frailty syndrome and cognitive decline, according to Relative Risk. Ribeirão Preto, SP, Brazil, 2018 *DC+ (Positive cognitive decline); †DC- = (Negative cognitive decline); ‡95% Confidence Interval

Figure 5 shows that four studies^(^ 19 ^,^ 22 ^,^ 25 ^-^ 26 ^)^ cross the vertical line of the graph, indicating lack of association between the variables. Considering RR (1.14) and 95%CI (0.96; 1.35), it is possible to affirm that the data did not show statistically significant association (p<0.13). *I* ^2^ (0%) indicates lack of heterogeneity with p (0.77) indicating that this result is not significant.

The quality assessment of the evidence, according to MINORS^(^ 16 ^)^, revealed a total value of 9.2 points, with variation from zero to two. The item unbiased assessment of the study endpoint was not described in the studies. Clearly stated objective were described in seven studies^(^ 19 ^-^ 20 ^,^ 22 ^,^ 24 ^-^ 27 ^)^; endpoints appropriate to the aim of the study were presented in nine studies^(^ 19 ^-^ 26 ^,^ 28 ^)^ and follow-up periods appropriate to the aim of the study were described by the authors of the 11 articles included^(^ 10 ^,^ 18 ^-^ 27 ^)^.

## Discussion

Aging is a gradual process characterized by individual and diverse trajectories. In a biological perspective, it is characterized by physical, cognitive and social alterations that lead to increased susceptibility to adverse health events^(^ 29 ^)^.

Aging is a process characterized by progressive, time-dependent, and heterogeneous decline in physiological function. It is orchestrated by a plethora of molecular mechanisms that change body homeostasis^(^ 30 ^)^, causing different geriatric syndromes, which, in turn, have a negative effect on quality of life and lead to an increase in disabilities and in the use of medical resources^(^ 31 ^)^.

Frailty syndrome is a new pathophysiological concept that has gained relevance, especially because it can be applied in clinical practice and is considered an important prognostic criteria for difficult therapeutic decisions^(^ 28 ^)^. This can be explained by the genetic and epigenetic factors, nutrient-sensing systems, mainly the so-called insulin signaling pathway, the growth factor, mitochondrial dysfunction, cellular senescence, stem cell exhaustion, inflammation, and some hormonal systems involved in the aging process^(^ 32 ^)^.

The accumulation of molecular and cellular damage in the aging process can cause hormonal and inflammatory dysregulation that leads to frailty and cognitive decline^(^ 33 ^)^.

Physical frailty and cognitive decline are frequent conditions among older adults. The association between physical frailty and cognitive decline may help identifying individuals with cognitive decline related to non-neurodegenerative causes, which may be reversible. In this sense, cognitive decline related to physical causes may be addressed in multidisciplinary interventions aimed at improving the quality of life of older adults.

In the literature, studies that address the topics of physical frailty and cognitive decline separately are more common. With the objective of clarifying the association between these two conditions and promoting new possibilities of research to support multidisciplinary interventions, the International Consensus Group, organized by the International Academy on Nutrition and Aging (I.A.N.A) and the International Association of Gerontology and Geriatrics (I.A.G.G) held a working group that defined the new concept of “cognitive frailty”. This new construct extends the definition of physical frailty by including the cognitive issue^(^ 34 ^)^.

Cognitive frailty is defined as a “heterogeneous clinical manifestation in older adults characterized by the simultaneous presence of both physical frailty and cognitive impairment”. In order to define such a condition, it is also necessary to exclude Alzheimer’s disease or other dementias^(^ 35 ^)^.

Among the studies included in the review, only one presented the definition of cognitive frailty as theoretical framework^(^ 22 ^)^. However, despite not applying the concept of cognitive frailty, other authors used its criteria (physical frailty, cognitive decline and absence of dementia) to evaluate the association between physical frailty and cognitive decline even though the construct of cognitive frailty has not been used as referential.

This decline is caused by white matter hyperintensities in the brain, related to small vessel injuries, breakdown of the blood-brain barrier and oxidative damage in brain tissue, which decreases the connections between different regions of the brain that can be seen in magnetic resonance imaging^(^ 6 ^,^ 36 ^)^.

The data suggest that physical frailty and cognitive decline have common pathophysiological mechanisms^(^ 26 ^,^ 37 ^-^ 38 ^)^.

Longitudinal studies on the temporal association between cognition and frailty found that the frailty domains are associated with lower performance in the cognitive domains, that frailty increases the risk of cognitive decline and dementia, and that there’s higher risk of mortality throughout follow-up of participants over time^(^ 39 ^)^_._

Decline in cognitive function is a process that occurs in the course of the aging process and is subject to multiple alterations that can lead the older person to develop some type of dementia^(^ 40 ^)^.

This data is confirmed by a study conducted in China with 19,943 participants over 65 years of age in a 12-year follow-up. The results showed greater cognitive decline among older adults living in rural area than among those living in urban area. This difference was associated with lower level of education, limited access to health services and decreased physical activity^(^ 41 ^)^.

The identification of the risk of cognitive decline related to physical causes becomes very relevant due to its potential for reversibility.

The results related to the association between frailty and cognitive decline were significant in a study with 2,737 older adults without cognitive impairment living in a community. Frailty was measured by the following aspects: reduction of skeletal muscle mass, grip strength and chair-stand test, weight loss, reduced walking speed and step length. The results indicated that for all men, all measures of frailty were associated with a decrease in the MMSE score over four years^(^ 24 ^)^.

Physical frailty and cognitive deficit are closely associated, and one component may affect the other and initiate a cycle of adverse events, such as functional disability, altered quality of life, dementia and death^(^ 38 ^)^.

The results of this systematic review showed a prevalence of the association between frailty and cognitive decline, especially when frailty was identified as a physical syndrome. Only one study^(^ 26 ^)^ did not find this association, since it presented OR=1.04; 95%CI [0.75;1.44]. The authors pointed out, as a limitation of the study, the inclusion of healthy participants at each follow-up assessment, which may have led to an underestimation of the values.

Researchers have found a close association between physical frailty and cognitive decline, suggesting there are common underlying mechanisms between them. In addition, studies have also found a strong connection with cardiovascular risk factors, chronic inflammation, nutritional problems, cerebrovascular accident, Alzheimer’s or other neurodegenerative disease^(^ 42 ^-^ 43 ^)^.

In this review, the meta-analysis for RR did not show evidence of association. However, the values obtained in the studies may not have been adjusted for other variables.

With the demands that arise with an ageing population and the new syndromes, such as cognitive frailty, it is necessary to elaborate preventive interventions that include physical activity, training exercises/cognitive stimulation and adoption of healthy eating habits.

Within a multidisciplinary team, nurses have a central role in all phases of care of these individuals. Nurses can facilitate the communication between professionals and family members, enabling a better comprehension and helping family members is to know more about the evolution, the possibilities of intervention and the prevention of future conditions, thus promoting a better quality of life for older adults^(^ 44 ^)^.

The relevance of the studies depends on their methodological quality, and the evaluation of this issue is considered important to guarantee the rigor of the systematic review. The methodological quality assessment or critical evaluation is the process of establishing internal validity, by verifying possible biases and the reliability of the evidence^(^ 15 ^)^. Most of the studies included in the present review show that the meta-analysis provides evidence on the association between these two variables: frailty and cognitive decline. Therefore, health professional must evaluate older adults using proper instruments to detect frailty syndrome in association with cognitive decline.

## Limitations

This systematic review has four limitations; two are related to the different operational definitions of frailty syndrome and cognitive decline in older adults, and the other two are related to values, variables and measures of association. Of the eleven studies included in this review, ten used the same operational definition of frailty syndrome (frailty phenotype), but even among those there was a significant variation in the items in the scale and their evaluation.

In the assessment of cognitive decline, the absence of standardization can be pointed as a limitation. At the same time, it is valuable to have different approaches in order to analyze which would be the best criterion to study the association of frailty syndrome and cognitive impairment. Another limitation of this review is the fact that the values obtained to calculate the RR were not adjusted for other variables, which may have influenced the result of the comparison between the studies.

In addition, the use of different measures of association in the studies (OR, HR, RR, mean, correlation) made it difficult to compare the results.

## Conclusion

All the eleven studies included in this review were observational studies with prospective follow-up, which assessed older adults at different times.

Despite of the methodological differences and different theoretical frameworks of the studies, this review evidenced the association between Frailty syndrome and Cognitive impairment. It is not possible to establish the defined proportion of the cause and effect relationship; however, most studies indicate Frailty syndrome as a trigger for Cognitive impairment.

These data are relevant when contextualized within the care model that focuses on health promotion and disease prevention, since it can support decision making in the planning of care for older adults. This way, preventive actions related to Frailty syndrome and Cognitive impairment contribute directly to the promotion of healthy aging.

This topic is very recent and deserves further research in epidemiological, clinical studies and in multicenter studies. In addition, the data may support the debate and the elaboration of public policies focused on Frailty syndrome and Cognitive impairment, in order to deal with the demographic growth of the country and the higher life expectancy of the population.
